# Symphony of epigenetic and metabolic regulation—interaction between the histone methyltransferase EZH2 and metabolism of tumor

**DOI:** 10.1186/s13148-020-00862-0

**Published:** 2020-05-24

**Authors:** Tengrui Zhang, Yueqing Gong, Hui Meng, Chen Li, Lixiang Xue

**Affiliations:** 1 Center of Basic Medical Research, Peking University Third Hospital, Institute of Medical Innovation and Research, 49 North Garden Road, Haidian District, Beijing, 100191 China; 2 Department of Radiation Oncology, Peking University Third Hospital, Beijing, 100191 China

**Keywords:** EZH2, Histone modification, Metabolism, Tumor therapy

## Abstract

Increasing evidence has suggested that epigenetic and metabolic alterations in cancer cells are highly intertwined. As the master epigenetic regulator, enhancer of zeste homolog 2 (EZH2) suppresses gene transcription mainly by catalyzing the trimethylation of histone H3 at lysine 27 (H3K27me3) and exerts highly enzymatic activity in cancer cells. Cancer cells undergo the profound metabolic reprogramming and manifest the distinct metabolic profile. The emerging studies have explored that EZH2 is involved in altering the metabolic profiles of tumor cells by multiple pathways, which cover glucose, lipid, and amino acid metabolism. Meanwhile, the stability and methyltransferase activity of EZH2 can be also affected by the metabolic activity of tumor cells through various mechanisms, including post-translational modification. In this review, we have summarized the correlation between EZH2 and cellular metabolic activity during tumor progression and drug treatment. Finally, as a promising target, we proposed a novel strategy through a combination of EZH2 inhibitors with metabolic regulators for future cancer therapy.

## Introduction

Lately, one of the exciting news in the field of epigenetic translational research is about EZH2. Tazemetostat (Tazverik; Epizyme), EZH2 inhibitor, has just been approved by the FDA for the first time for the treatment of adults and adolescents with locally advanced or metastatic epithelioid sarcoma in Jan 23, 2020 [1, 2]. Looking back the research history regarding EZH2, although it has been found closely correlated with majority of tumor genesis, metastasis, and prognosis in decades, the effectiveness in previous clinical trials targeting EZH2 was not satisfactory, especially in solid tumor. To further improve the clinical outcome of EZH2 inhibitors, substantial in-depth investigation still needs to be performed to gain the comprehensive understanding of epigenetic regulation mediated by EZH2 in tumor cells.

Besides the epigenetic dysregulation, tumor cells also present very distinct characteristics of metabolism to meet the massive materials and energy demands for the rapid proliferation. For instance, a considerable proportion of tumor cells assimilate high levels of glucose and produce lactic through glycolysis which termed as the “Warburg effect” [3]. Tumor cells often upregulate the de novo synthesis or uptake of fatty acids to adapt to vigorous demand of lipids [4]. Glutamine is consumed at high rates by various types of tumor cells in order to support energy production and biosynthesis [5]. Targeting the key metabolic process has become another promising strategy to conquer the tumor.

Emerging evidences has revealed that EZH2 is involved in metabolic reprogramming by canonical and uncanonical manner; in turn, several metabolites and metabolic processes offer the donors for EZH2 post-translational modification. Thus, the transcriptional regulation from EZH2 and the post-translational modification derived from metabolism formed a dynamic crosstalk which serves as the more precise regulatory network. Therefore, we reviewed the latest proceedings in this field and aim to provide the insight in potential combination approach to fight against tumors.

## EZH2 promotes tumorigenesis and progression epigenetically

Epigenetic modifications can regulate chromatin state and gene expression through DNA methylation and demethylation, histone modification, chromatin remodeling, and other processes without altering the DNA sequences [6, 7]. Polycomb group proteins (PcGs), which include polycomb repressive complexes 1 and 2 (PRC1 and PRC2), are master epigenetic regulatory factors involved in embryogenesis, stem cell differentiation, chromatin modification, and tumor progression [8, 9]. EZH2 is the core and catalytic subunit of PRC2, which catalyzes the trimethylation (H3K27me3) of histone H3 lysine 27 through its C-terminal SET domain, thus promoting chromatin compression and gene silencing [10, 11].

Abnormal functioning of EZH2 is closely associated with tumorigenesis and progression. Early evidence has confirmed that the overexpression of EZH2 is associated with the worsening of prostate cancer progression [12]. Similarly, subsequent studies have widely implicated the overexpression of EZH2 in the development of various solid malignancies including those of prostate [12], breast [13], bladder [14] and pancreatic cancers [15], hepatocellular carcinoma [16], and melanoma [17] among others. High levels of EZH2 indicate a strong correlation with tumor aggressiveness and poor prognosis in many types of cancers.

Furthermore, it has been found that EZH2 is frequently mutated in certain hematological malignancies demonstrating gain or loss of function. For example, in 22% of the germinal central B cell (GCB) subtype of diffuse large B cell lymphoma (DLBCL) and in 7% to 12% of follicular lymphoma (FL), a recurrent heterozygosity mutation is observed in the tyrosine (Y641) of the SET domain of EZH2 [18, 19]. These mutations can enhance the function of EZH2 by trimethylating H3K27, ultimately inhibiting the expressions of genes associated with differentiation in lymphoma (such as *TCF4*, *FOXP1*, and *CDKN1A*) [20–23]. However, in cases of myelodysplastic syndrome (MDS) [24], myeloproliferative tumor (MPN) [25], and myelodysplastic/myeloproliferative neoplasms (MDS/MPN) [26], it has been found that loss-of-function mutations in EZH2 could lead to the upregulation of certain oncogenic genes.

**Fig. 1:**
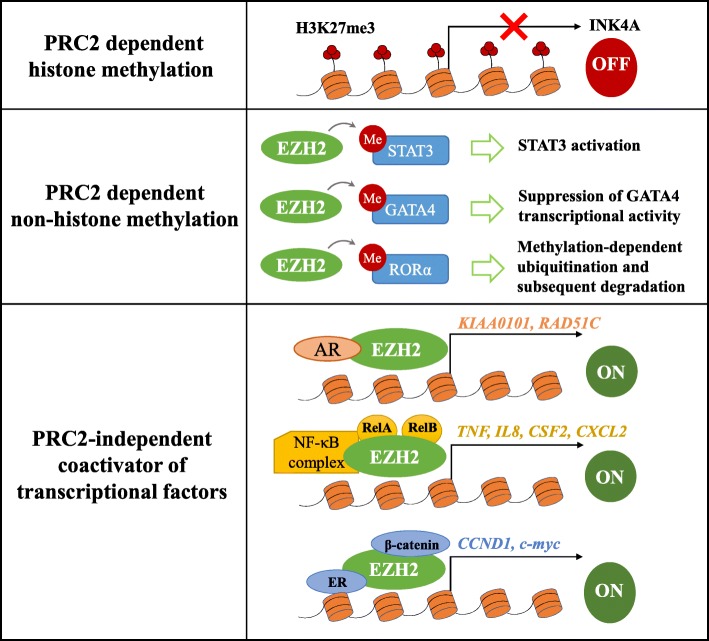
The mechanism of EZH2 in promoting tumorigenesis. (1) EZH2 methylates Histone 3 on lysine 27 depend on PRC2, which contributes to transcriptional silencing. (2) EZH2 is also capable of methylating some non-histone protein substrates which include STAT3, GATA4, and RORα. (3) EZH2 also can act as a coactivator of transcription factors in a PRC2-independent manner, such as AR, NF-κB complex, and ERα

EZH2 can promote tumorigenesis through various mechanisms, which are as follows (Fig. 1):

1. PRC2-dependent histone methylation: This is the canonical mechanism of EZH2. For example, transcriptionally silencing tumor suppressor genes such as *Ink4A/Arf* through EZH2-mediated trimethylation of H3K27 could result in an uncontrolled progression of the cell cycle [27].
2. PRC2-dependent non-histone protein methylation: Besides canonical histone methylation, emerging evidence has indicated that non-histone proteins can also serve as substrates for EZH2. For example, in glioblastoma (GBM) [28] and melanoma [29], EZH2 mediates the lysine methylation of STAT3, leading to its activation, which enhances tumorigenicity. To date, studies have been conducted on non-histone substrates like STAT3, GATA4, and RAR-related orphan receptor α (RORα) [30].
3. PRC2-independent coactivator of transcriptional factors: It has been reported that EZH2 may act as a coactivator for the transcriptional factor androgen receptor (AR) to promote the expression of genes related to tumor cell growth in castration-resistant prostate cancer (CRPC) [31]. Additionally, in ER-negative basal breast cancer, EZH2 activates NF-κB and binds to a set promoter regions by forming ternary complexes with Rel A and Rel B to promote target gene expression and tumorigenesis [32].

Currently, the role of EZH2 in the pathogenesis and development of malignant tumors has been studied extensively. However, its underlying mechanism remains to be fully elucidated. Abnormal metabolic status is a key factor in the development and progression of tumors. Recently, evidence has suggested that EZH2 might be playing an important role in regulating cell metabolism. Therefore, EZH2 can influence the development and progression of tumors by interfering with cellular metabolic activities.

## EZH2 mediates carcinogenic effects via metabolic pathways

The metabolic characteristics of tumor cells, which are responsible for the massive requirement of nutrients and energy for their survival and proliferation, are different from those of normal cells. Epigenetic control can regulate the expression of genes involved in metabolism and change the metabolic profile of cells. Being one of the key factors involved in epigenetic regulation, EZH2 may regulate the metabolic activities of tumor cells, thereby affecting cancer progression.

### Metabolic characteristics of cancer cells

Tumorigenesis and progression are associated with the reprogramming of cellular metabolism driven by oncogenic mutations and microenvironmental factors. Metabolic reprogramming in tumors occurs in the metabolic pathways of glucose, amino acids, and lipids, as a result of which, metabolites required for anabolic processes are supplied in response to different stimuli and stress conditions favoring tumor development [33].

#### Metabolic requirements of tumor cells

Tumor cells need to consume massive nutrients (including glucose, amino acids, and fatty acids) to meet material and energy demands. Especially tumor cells in a rapidly proliferating state must undergo active biosynthesis to build blocks for the assembly of various macromolecules [33]. During tumor initiation or progression, even in aerobic environments, a considerable proportion of tumor cells cancer cells assimilate high levels of glucose and produce lactic acid through glycolysis, by a phenomenon known as the “Warburg effect” [3]. At the same time, although Warburg hypothesized that cancer cells adopt a glycolytic phenotype due to disruption of mitochondrial activities at OXPHOS level, mitochondria is still functional in cancer cells and retain the ability to conduct oxidative phosphorylation [33, 34]. As a result, tumor cells can adapt to fluctuating conditions of oxygen availability and can provide sufficient energy. Furthermore, tumor cells use intermediates of the glycolysis/TCA cycle to biosynthesize lipids, amino acids, and nucleotides, and generate NADPH [33]. For example, the intermediate metabolite glucose-6-phosphoric acid can enter the pentose phosphate pathway facilitating the production of NADPH and ribose-5-phosphoric acid [35], which provides the hydrogen and ribose-5-phosphoric acid for the synthesis of biomolecules and nucleotides, respectively.

In addition to glucose metabolism, metabolic reprogramming in tumors also occurs in the metabolic pathways of amino acids. Glutamine is consumed at high rates by various types of tumor cells in order to support energy production and biosynthesis [5]. Glutamine may serve as a source of energy for tumor cell and act as a nitrogen donor. Ingested glutamine can be used as a substrate allowing uptake of some essential amino acids. Intracellularly loaded l-glutamine is effluxed via hLAT1 (human L-type amino acid transporter 1) in exchange for extracellularly applied essential amino acids such as leucine and methionine [36]. Additionally, the decomposition product of glutamine can be converted into α-ketoglutarate, which enters and replenishes the TCA cycle. By contributing to citrate production, glutamine can also directly supply acetyl-CoA for lipogenesis [37].

Tumor cell metabolic reprogramming also affects lipid biosynthesis. Tumor cells often upregulate the *de novo* synthesis or uptake of fatty acids to adapt to vigorous demand for lipids. Lipid accumulation accelerates the formation of biofilm structures and resists lipid peroxidative damage caused by reactive oxygen species [4]. Additionally, the structural components of lipid raft within cell membrane are modulated by lipid metabolism. The specific structure domain mediating lipid signaling also contributes to tumor metastasis [38]. With the support of the above metabolic processes, metabolic reprogramming can directly contribute to tumorigenicity and malignancy [33].

#### Metabolic heterogeneity and adaptability of tumor cells

In fact, the properties of tumor microenvironment are not uniform. There are often some harsh subregions in tumors, in which tumor cells encounter metabolic stress, which includes hypoxia, acidification, nutrient deficiency, and accumulation of metabolic waste. In addition, there are great differences in the microenvironment of diverse types of cancer, such as the abundance of various metabolites and other soluble factors, as well as the proportions of malignant cells and other cell types [39]. The characteristics of the quantity and proportion among different components in the tumor microenvironment inevitably affect the metabolic status of tumor cells and the other cells [40]. For example, the content of oxygen and nutrients in the microenvironment can change the balance between the endogenous synthesis and exogenous uptake of fatty acids in tumor cells. Under the condition of sufficient nutrients and a high oxygen level, tumor cells tend to utilize glucose-derived acetyl-CoA for *de novo* synthesis of fatty acids [40]. However, the circumstance is different in hypoxic condition. Glucose is difficult to be converted into acetyl-CoA into the TCA cycle due to the inhibition of mitochondrial pyruvate dehydrogenase complex activity [41]. Therefore, tumor cells tend to enhance the intake of exogenous fatty acids as well as utilize other metabolites (such as glutamine and acetate) for synthesis of fatty acids. Besides, if oxygen and nutrient (such as fatty acids and glucose) are both deprived in the tumor microenvironment, tumor cells will mainly rely on the utilization of glutamine, acetate, and other substances for fatty acid synthesis. These evidence demonstrates that tumor cells have adaptability to various metabolic stress [40].

It is worth noting that the tumor metabolism is also closely related to the heterogeneity between different types of cells in the microenvironment. For instance, in addition to the Warburg effect, there is also a reverse Warburg effect in the tumor microenvironment. Tumor cells can promote the glycolysis process of some adjacent stromal cells (such as cancer-associated fibroblasts) to supply massive metabolites including lactate and pyruvate [42]. These metabolites can efficiently support tumor cells to maintain high levels of oxidative phosphorylation, resulting in tumor cells being more aggressive [43].

In conclusion, although tumor cells have certain commonalities in metabolism, their metabolic processes are affected by varied factors in the intracellular space and the microenvironments which are highly heterogeneous and dynamic through the whole life-span. Tumor cells adapt to metabolic pressure through the regulation network of metabolic activities.

### EZH2 regulates metabolism in cancer cells

In a study of investigating the relationship between PRC repression markers and RNA polymerase II (RNAPII) in mouse ESCs, Brookes et al. identified the four major PRC-target gene groups using ChIP-seq approach: PRC only, PRC repressed, PRC intermediate, and PRC active. Among PRC active target genes, the glycolysis and pyruvate related genes, such as *HK1*, *ENO2*, and *PCK2*, were found unexpectedly [44]. Besides the stem cells, the increasing evidence has proved that EZH2, being a catalytic subunit of PRC2 plays a key role in metabolic process in cancer cells as well, directly or indirectly.

#### EZH2 facilitates glucose metabolism in cancer cells

Studies have shown that EZH2 may promote aerobic glycolysis in tumors. In GBM cells, EZH2 can convert mitochondrial respiration to glycolysis in vitro by increasing the level of H3K27me3 in the EAF2 promoter region. This blocks the transcription of EAF2 and activates the HIF1α signaling pathway inducing the transcription of downstream genes involved in metabolism such as *HK2*, glucose transporter 1 (*GLUT1)*, and *PDK1*. Therefore, it can be stated that EZH2 can facilitate tumorigenesis and the malignant progression of tumor cells through induction of the Warburg effect [45].

EZH2 can also indirectly induce aerobic glycolysis in cancer cells by suppressing the expression of some microRNAs. Some metabolism-associated miRNAs are silenced by a repressive chromatin structure involving H3K27me3 mediated by EZH2 [46, 47]. In prostate cancer (PCa) cells, a group of genes related to glucose metabolism such as those of the key glycolytic enzyme *HK2*, *GLUT1*, and ribosomal protein S6 kinase B1 (*RPS6KB1*) are positively correlated with the expression of EZH2 [46]. *HK2* is one of the downstream target genes of miR-181b. EZH2 inhibits the expression of miR-181b through increasing its H3K27me3 level, and thus indirectly upregulates the expression of HK2. As a result, EZH2 promotes glucose metabolism and facilitates tumor cell survival [46]. In glioma cells, inhibition of the EZH2 activity suppresses aerobic glycolysis. It is revealed that the glycolytic capacity and reserve are both diminished when the levels of EZH2 are decreased in U87 and U251 glioma cells. EHZ2 can bind to miR-328 promoter and downregulate miR-328 by a canonical H3K27me3 modification manner. Meanwhile, miR-328 is found to inhibit the expression of β-catenin. This EZH2/miRNA/β-catenin feed-forward loop leads to an increase in the extracellular acidification rate (ECAR) implying an enhancement in the glycolytic ability [47]. In summary, it can be stated that EZH2 can promote glucose metabolism in tumor cells, thus maintaining and promoting their high energy demands and survival, respectively.

#### EZH2 promotes lipid synthesis in cancer cells

EZH2 is an important regulator of lipid metabolism. Abundant evidence has indicated that the methyltransferase activity of EZH2 is necessary for adipogenesis. Severe defects in adipogenesis in Ezh2^−/−^ mouse primary preadipocytes have been reported [48]. It has been determined that, in mouse white fat cells, adipogenic stimuli triggers the nuclear translocation of S6K1, leading to the phosphorylation of H2BS36 and recruitment of EZH2 to H3, resulting in the trimethylation of H3K27. This represses the expression of the *Wnt* gene and induces the upregulation of PPARγ and C/EBPα to facilitate adipogenesis [49, 50]. It has also been reported the synthesis of triglycerides in the liver cells was upregulated by deacetylated SREBP1-1c mediated by SIRT6, while SIRT6 can be silenced by EZH2 and rescued by the upstream lncRNA PU.1 AS. Overall, EZH2 is known to facilitate adipogenesis significantly [51].

It is known that EZH2 also promotes lipid synthesis in cancer cells. In glioma harboring telomerase reverse transcriptase (TERT) promoter mutations, TERT and EZH2 cooperate in the activation of PGC-1α, which is involved in the expression of fatty acid synthase (FASN). Pharmacological inhibition of human TERT represses the expression of EZH2 and FASN and decreases the accumulation of fatty acids. Conversely, diminished expression levels of TERT and FASN, as well as reduced abundance of intracellular fatty acids, are observed upon siRNA-mediated EZH2 knockdown. Elevated EZH2 levels in TERT mutants play a fundamental role in gliomagenesis through epigenetic reprogramming of H3K27me3 modification. Knocking down EZH2 not only affects TERT expression but also lipid metabolism. Thus, it can be elucidated that EZH2 promotes the synthesis of fatty acid and lipid accumulation through the TERT-EZH2 network. However, the comprehensive mechanism by which EZH2 regulates lipogenic genes (such as *PPARGC1A*, *FASN*) still needs to be investigated [52]. Moreover, it has been reported that high levels of fatty acids in cancer cells can negatively regulate the expressions of p21, Bax, and p53, which participate in the DNA damage repair (DDR) pathway [53]. This results in the inhibition of apoptosis, leading to tumor progression.

However, several studies have shown that the inhibition of EZH2 induces lipid accumulation in hepatocyte cell lines [54] and certain cancer cells such as breast cancer [55]. To address this discrepancy, Yiew et al. [56] investigated the role of EZH2 in adipogenic differentiation and lipid metabolism using primary human and mouse preadipocytes and adipose-specific EZH2 knockout (KO) mice. They found that the inhibition of EZH2 could induce lipid accumulation in adipocytes by reduction in H3K27me3 modifications and upregulating expression of the *APOE* gene in adipocytes rather than affecting adipogenic differentiation, which was contrary to the previous elucidation that EZH2 is a positive regulator of adipogenic differentiation observed in murine adipose progenitor cells [48]. This discrepancy may have arisen due to differences in the adipocyte progenitor cell lineages and/or species. In summary, the potential mechanisms by which EZH2 regulates lipid metabolism remain to be investigated.

#### EZH2 is involved in amino acid metabolism in cancer cells

The regulation of amino acid metabolism by EZH2 is mainly reflected in two aspects, which are as follows: (1) inactivated mutation of EZH2 can upregulate glutamine metabolism, which fuels the tricarboxylic acid (TCA) cycle, nucleotide and fatty acid biosynthesis, and redox balance in cancer cells [5, 57], and (2) EZH2 promotes the synthesis of s-adenosine methionine (SAM), which alters the epigenetic patterns by affecting the transport of one carbon unit [58].

**Fig. 2:**
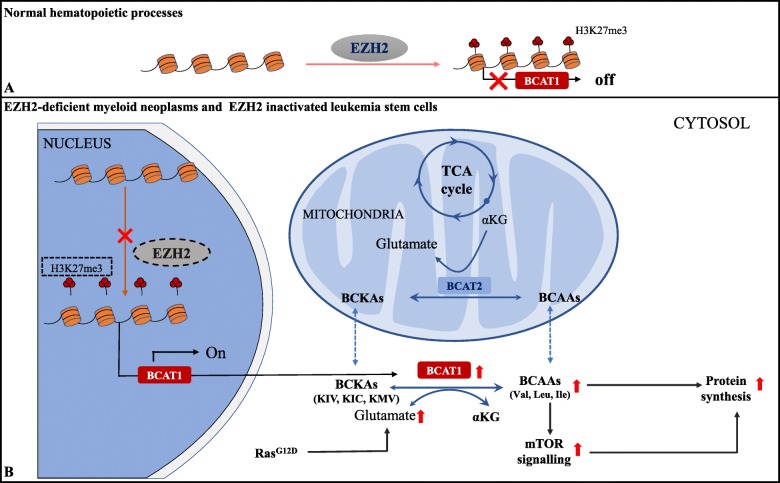
EZH2 regulates glutamine metabolism by silencing BCAT1 transcription through catalyzing H3K27me3. **a** BCAT1 is repressed by EZH2-PRC2 mediated H3K27me3 in normal hematopoietic processes. **b** BCAT1 is abnormally activated in EZH2-deficient myeloid neoplasms and EZH2 inactivated leukemia stem cells

It is known that glutamine metabolism increases in tumors with EZH2 inactivated mutations, and the activated energy metabolism promotes tumor progression [57, 59]. BCAT1 is a branched-chain amino acid transferase isoenzyme, which catalyzes the reversible transamination of branched-chain amino acids (BCAAs). Reversible transamination results in transfer of the amidogen from BCAA to α-ketoglutarate (α-KG), thus generating glutamate and the corresponding branched-chain α-keto acids (BCKAs) [59]. BCAT1 is repressed by EZH2 via H3K27me3 modification in normal hematopoietic processes but is abnormally activated in EZH2-deficient myeloid neoplasms in mice and humans. BCAT1 reactivation along with NRAS^G12D^ mutations can sustain intracellular BCAA pools, resulting in enhanced mTOR signaling in EZH2-deficient leukemia cells [60]. Similarly, in EZH2 inactivated leukemia stem cells (LSC), the combined effect of the loss of EZH2 and NRAS enhance BCAT1-mediated BCAA metabolism and upregulated the expression of genes involved in TCA (e.g., *IDH*) [57] (Fig. 2). Interestingly, BCAT1 is also repressed by G9a/SUV39H1-mediated H3K9me2 and H3K9me3. In EGFR mutant lung cancer cells, H3K9 demethylation mediated upregulation of BCAT1 and subsequent metabolic reprogramming, which promotes TKI (lethal EGFR tyrosine kinase inhibitors) resistance through attenuating reactive oxygen species (ROS) accumulation [61].

**Fig. 3:**
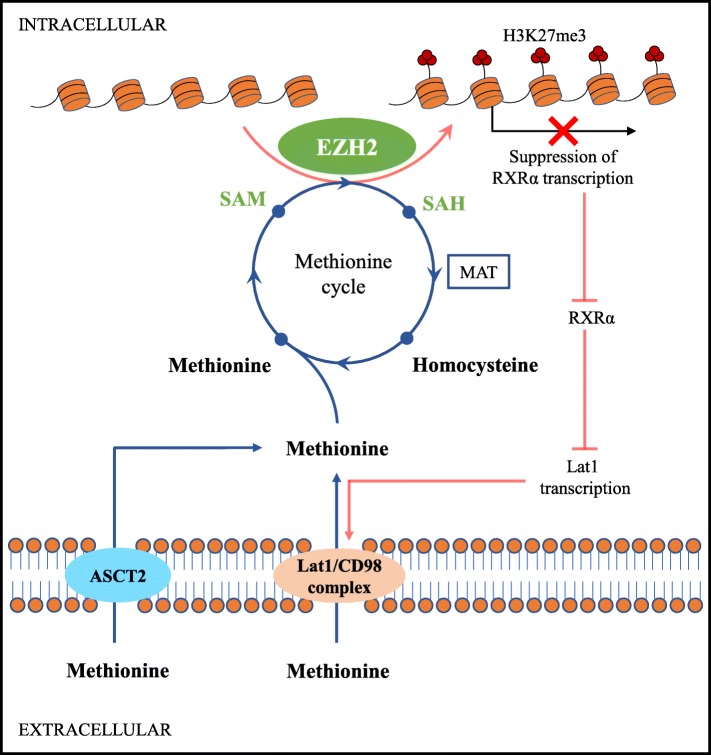
EZH2/Lat1 positive feedback loop promotes the generation of SAM. EZH2 derepresses Lat1 expression and promotes the generation of SAM through direct promoter binding and subsequent transcriptional repression of RXRα

EZH2 may also regulate amino acid metabolism by promoting SAM synthesis. Methionine is a precursor of SAM and its cycle flux specifically influences the methylation of DNA and histones in cancer cells and drives tumor initiation [62]. Lat1 (SLC7A5) is an amino acid transporter often required for the import of essential amino acids, including methionine, in tumor cells [63]. It has been found that knocking down EZH2 or inhibiting its methyltransferase activity can result in the activation of retinoid X receptor α (RXRα) genes, which inhibit the transcription of Lat1 in lung cancer H1299 cells [58]. Consequently, the highly active EZH2/Lat1 positive feedback loop promotes the generation of SAM, which enhances the histone methyltransferase activity of EZH2 and accelerates tumor progression (Fig. 3).

## Effect of tumor cell metabolic reprogramming on EZH2 function

Metabolic activities may affect EZH2 function by two known mechanisms, which are as follows: (1) metabolism directly changes the methyltransferase activity of EZH2 by regulating the level of SAM, and (2) small molecules generated because of metabolic activities serve as donors of chemical groups used for posttranslational modifications, leading to the alteration of EZH2 function and stability of PRCs. These modifications include phosphorylation, acetylation, ubiquitination, sumoylation, and O-linked N-acetylglucosamine modification (O-GlcNAcylation) [64]. Thus, EZH2 can regulate nutrient uptake and utilization and cell metabolism can affect its function.

### Effect of metabolism on the activity of EZH2 methyltransferase

#### SAM/SAH ratio is a major determinant of histone methylation

SAM is the methyl donor in methyltransferases, which is catalyzed by methionine adenosyl transferase (MAT) using methionine and ATP as substrates. One carbon metabolism links products from multiple metabolic pathways such as glucose, serine, threonine, methionine, and choline [65]. S-adenosyl-l-homocysteine (SAH) is the product formed after SAM releases the methyl group and is an effective inhibitor of methyltransferase (DNMT and HMT) [66, 67]. Therefore, the SAM/SAH ratio serves as the main indicator of histone methylation [68, 69]. In human pluripotent stem cells, methionine deprivation reduces the methylation of histones in which the level of H3K4me3 decreases and promotes the differentiation of pluripotent stem cells [70]. It has been reported that histone methylation (H3K4me3, K9me3, K27me3) has significantly reduced when SAM production is restricted by methionine deprivation in HCT116 colorectal cancer cells or C57Bl6 mice. It is found that the methionine cycle (including SAM, SAH, and other substances) and histone methylation kinetics respond to methionine limitation under conditions of methionine deprivation at different concentrations and periods. Subsequently, the levels of SAM, SAH, and histone methylation are found to decrease [68]. In tumors, an increased SAM/SAH ratio is linked with the hypermethylation of tumor suppressor gene silencing, while its decrease helps reduce the methylation of oncogene promoters [68]. For example, the SAM/SAH ratio and histone methylation (H3K4me3, K9me3, and K27me3) were found to be significantly reduced after methionine deprivation for 24 h in the colorectal cancer cells HCT116. ChIP-seq analysis revealed that H3K4me3 in the promoter regions of oncogenes (such as *AKT1*, *MYC*, and *MAPK*) had decreased significantly [68].

#### SAM inhibitors are potential antitumor candidates

Compared to H3K4me3 and H3K9me3, H3K27me3 is not the most sensitive to changes in the SAM/SAH ratio. However, it has been reported that suppressing the activity of SAH hydrolase (such as DZNep) or hindering the linkage of EZH2 with SAM can significantly downregulate the global H3K27me3, leading to the inhibition of tumor cell (such as breast, bladder, and lung cancers) survival [71, 72]. Histone methyltransferases utilize SAM to methylate their substrates and this is a reversible reaction. Accumulation of SAH inhibits the activity of histone methyltransferases (HMT). Inhibition of SAH hydrolase leads to the accumulation of SAH in MCF7 breast cancer and T24 bladder cancer cells, which finally inhibits the HMT activity of EZH2 and downregulates the level of H3K27me3 [73]. Many highly efficient and selective EZH2 inhibitors have been identified, which include EPZ005687 [74], GSK343 [75], and GSK126 [76]. All of these impair the activity of methyltransferase by competing with SAM to bind EZH2. Therefore, targeting SAM and SAH, products of the one-carbon metabolism cycle, is a promising novel strategy that can be used for cancer therapy. Additionally, α-ketoglutarate (α-KG), an intermediate of the TCA cycle, is an essential cofactor of DNA demethylase (TET) and histone demethylase (JMJD), which may also affect histone methylation [77]. Changes in the levels of αKG may affect the modification level of H3K27me3. However, no evidence suggesting the direct involvement of αKG in EZH2 function is available to date.

### Effects of metabolites on post-translational modification of the EZH2 protein

#### Phosphorylation downregulates the enzyme activity of EZH2 and stability of the PRC2 complex

EZH2 is phosphorylated by various kinases, including AMP-activated protein kinase (AMPK) [78], AKT [79], CDK1 [80], CDK2 [81], and p38 [82]. EZH2 phosphorylation is majorly associated with the inhibition of its methyltransferase activity, leading to a decrease in the level of H3K27me3 [78, 79, 83]. Interestingly, phosphorylation at different sites of EZH2 is associated with distinct biological functions. EZH2 is phosphorylated on Thr311 by AMPK, and on Ser21 by AKT. Both the kinases are closely linked to the regulation of energy metabolism. Phosphorylation by AMPK and AKT can both downregulate the enzymatic activity of EZH2, but mediate tumor-suppressive effects and promote tumor progression in different cellular context, respectively.

AMPK coordinates many cellular processes (including metabolism, protein synthesis, and autophagy) to maintain energy homeostasis [84, 85]. Persistent energy deprivation leads to the activation of AMPK. It has been reported that cellular energy stress (such as glucose deprivation and glycolysis block) can activate AMPK kinase in human breast and ovarian cancer cells. This results in the phosphorylation of EZH2 at Thr311 disrupting its interaction with SUZ12. Consequently, the HMTase activity of PRC2 is found to be inhibited, thereby relieving the repression of target genes resulting in cell cycle arrest and cell differentiation. Moreover, the activity of AMPK was positively correlated with the presence of pT311-EZH2 in clinical samples of ovarian and breast cancer. Patients with higher levels of pT311-EZH2 had better survival rates against ovarian and breast cancers [78]. This is the most direct evidence proving that tumor metabolism affects the enzymatic activity of EZH2 via post-translational modification (PTM).

Akt is a serine/threonine kinase involved in the regulation of cell survival growth, proliferation, migration, metabolism, and angiogenesis and plays a key role in energy metabolism regulation [86, 87]. In tumors, Akt increases the expression of glucose transporters GLUT1 and GLUT4 and activates enzymes involved in glycolysis such as HK and PFK1/2 (phosphofructokinase 1/2), thereby enhancing the viability of tumor cells undergoing aerobic glycolysis [88]. AKT may phosphorylate EZH2 under certain conditions. First, Akt-mediated pS21-EZH2 inhibits its HMTase activity by impeding the binding of EZH2 to histone H3 instead of changing its subcellular localization or interaction with other PcG proteins such as SUZ12 and EED [79]. It was revealed that insulin-like growth factor (IGF) activates Akt in MDA-MB453 cells, which phosphorylates EZH2 at Ser21 (pS21-EZH2) leading to a decrease in the levels of H3K27me3. Consequently, decreased amounts of H3k27me3 release the EZH2 silenced genes (such as *HOXA9*) and promote tumor progression. Additionally, the presence of Akt-dependent pS21-EZH2 is also observed in multiple myeloma (MM) cells, which interact with bone marrow stromal cells (BMSCs) containing markers such as SDF1, IGF1, and FN1, resulting in reduced activities of H3K27me3 and HMTase [83]. As a result, the derepression of antiapoptotic genes (*IGF1*, *CXCL2*, *BCL2*, and *HIF1α*) leads to drug resistance in MM cells. Second, pS21-EZH2 by PI3K/AKT signaling is observed as a transcriptional coactivator in CRPC in a PRC2-independent manner, promoting the expression of cyclin-D2 and cell growth [31]. Third, Akt-mediated pS21-EZH2 can also contribute to tumorigenicity by methylating crucial non-histone substrates. In glioblastoma stem-like cells (GSC), Akt-induced pS21-EZH2 can facilitate EZH2-STAT3 interaction, enhancing EZH2-mediated methylation and activity of STAT3, which accelerates GSC self-renewal and glioblastoma tumor progress [28].

The effect of metabolic stress on AKT is still controversial. AKT activity differs among cancer cells under conditions of glucose deprivation [89]. For example, AKT is activated in HeLa cells but is inhibited in those of ovarian cancer. In HeLa cells, glucose deprivation induces AKT phosphorylation at both Thr308 and Ser473, leading to cell survival during metabolic stress [90]. However, AKT is relatively activated when glucose is abundant in ovarian cancer cells, promoting their growth, division, and metastasis. Studies have determined that, upon glucose deprivation, AMPK is activated while AKT phosphorylation is suppressed, inhibiting AKT-mediated apoptosis [91]. The effect of tumor metabolism on AKT has not yet been fully elucidated. Whether change in the metabolic profile can influence AKT and its phosphorylation on EZH2 requires further investigation.

#### Acetylation enhances the stability and function of EZH2

Acetylation of EZH2 requires acetyl-CoA, which is synthesized in the mitochondria and nucleus. In the mitochondrion, acetyl-CoA is produced through glycolysis and fatty acid β-oxidation. Glycolysis and degradation of specific amino acids yield pyruvate, which can be oxidized to acetyl-CoA by the pyruvate dehydrogenase complex (PDC). In the nucleus, acetyl-CoA is synthesized from acetate and citrate using acyl-CoA synthetase short-chain family member 2 (ACSS2) and ATP-citrate lyase (ACLY) [92, 93]. Wan et al. [94] have found that EZH2 can be acetylated and deacetylated by the acetyltransferase P300/CBP-related factor (PCAF) and deacetylase SIRT1, respectively. PCAF can directly interact with EZH2 and acetylate EZH2 mainly at lysine 348 (K348) in vitro and in vivo. K348 acetylation reduces EZH2 phosphorylation at T345 and T487 and enhances EZH2 stability without disrupting its interaction with SUZ12 and EED or localization. EZH2 K348 acetylation is important for silencing its target genes and enhancing the migration and invasion abilities of lung cancer cells in vitro. Lung cancer tissues demonstrate increased levels of EZH2 acetylation at K348 compared to those observed in normal lung tissues. Patients with a high level of EZH2 K348 acetylation correlate with poor prognosis.

#### O-GlcNAcylation enhances the stability and function of EZH2

O-GlcNAcylation is involved in many cellular processes, including transcription, signal transduction, and apoptosis [95]. O-GlcNAcylation is a post-translational modification, which adds O-GlcNAc to the Ser or Thr residues of a given protein. It is mediated by O-GlcNAc transferase (OGT) and O-GlcNAcase (OGA) [96]. In the process of tumorigenesis and tumor development, O-GlcNAcylation is closely related to the functions of many transcription factors, tumor suppressor genes, proto-oncogenes, and cell receptors [97]. The regulation of EZH2 by O-GlcNAcylation was first reported in breast cancer cells. It was found that EZH2 is O-GlcNAcylated in an OGT-dependent manner at serine 75 (S75) and this modification enhances the stability of EZH2, leading to continuous activation of H3K27me3 and promotion of tumorigenesis [98]. Four additional O-GlcNAcylation sites of EZH2 (including S73, S84, T313, and S729) have been identified in OGT overexpressed 293T cells by liquid chromatography-electrospray ionization-mass spectrometry (LC-ESI-MS) [99]. In this OGT ectopic expressed 293T cells, mutation in one or more of the four sites (S73, S76, S84, and T313) decreases the stability of EZH2 but does not affect its binding with SUZ12 and EED. Mutation at S729 has eliminated the dimethylation and trimethylation of H3K27. However, this is not found to alter its monomethylation or impair the integrity of the PRC2/EZH2 core complex [99]. O-GlcNAcylated EZH2 have downregulated the epithelial cell markers claudin-7 and E-cadherin and enhanced tumor migration and invasion, leading to the metastasis of advanced colorectal cancer (CRC) [100]. It has been reported that microRNA-101 can inhibit the expression of OGT and EZH2 in CRC. In addition, O-GlcNAcylation and H3K27me3 modification in the miR-101 promoter region further inhibited the transcription of miR-101, resulting in the upregulation of OGT and EZH2 in metastatic CRC, thus forming a vicious cycle. This negative-feedback loop leads to the upregulation of OGT and EZH2 in metastatic CRC eventually resulting in tumor development.

**Fig. 4:**
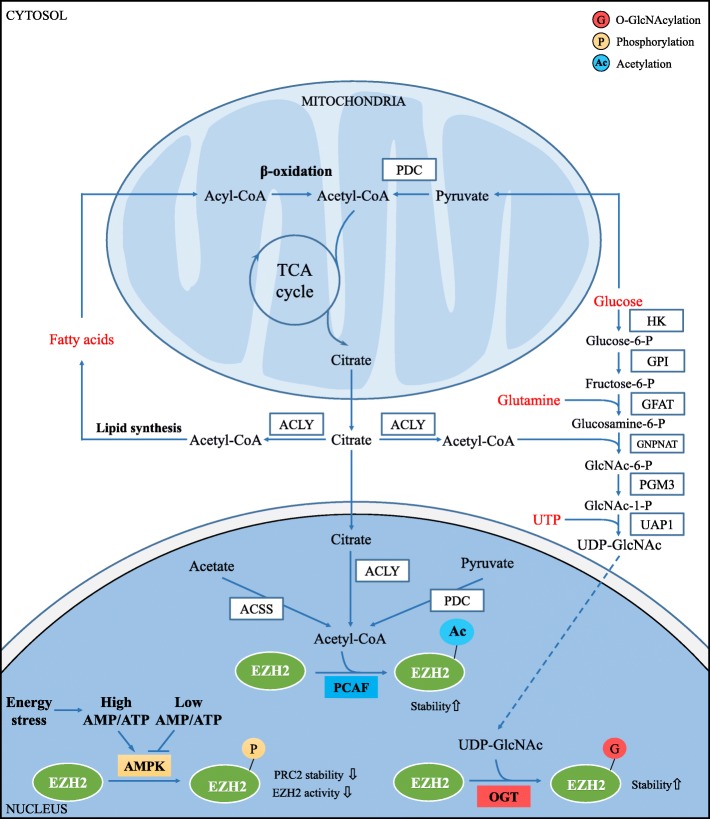
Effects of metabolites on post-translational modification of EZH2 protein. Post-translational modification of EZH2 requires metabolites to participate. EZH2 acetylation by P300/CBP-related factor (PCAF) uses the metabolite acetyl-CoA, enhancing the stability of EZH2. Acetyl-CoA is synthesized in the cytoplasm and nucleus from acetate, citrate, or pyruvate by acyl-CoA synthetase short-chain family member 2 (ACSS2), ATP-citrate lyase (ACLY), and pyruvate dehydrogenase complex (PDC), respectively. EZH2 GlcNAcylation is mediated by O-GlcNAc transferase (OGT) utilizing UDP-glucosamine (UDP-GlcNAc) which is generated from the hexosamine biosynthetic pathway. The stability and function of EZH2 is enhanced by O-GlcNAcylation modification. Energetic stress can affect EZH2 phosphorylation by activating AMP-activated protein kinase (AMPK), leading to a decrease in PRC2 stability and EZH2 activity

O-GlcNAcylation is dependent on the metabolites of the hexosamine biosynthetic pathway (HBP), which interacts with the glucose, amino acid, fatty acid, and nucleotide metabolic pathways. The final product of the HBP pathway is uridine diphosphate GlcNAc (UDP-GlcNAc), a donor of O-GlcNAcylation [101]. Since the biosynthesis of UDP-GlcNAc requires glucose, glutamine, acetyl-CoA, and UTP, the quantity of these substances can affect intracellular O-GlcNAcylation. Therefore, the levels of O-GlcNAcylation reflect the dynamic changes occurring in the metabolic processes. For example, hyperglycemia may increase O-GlcNAcylation and induce high-glucose-stimulated liver tumorigenesis in a YAP-dependent manner [102] (Fig. 4 and Table 1).

**Table 1:** Effects of metabolites on post-translational modification of EZH2 protein

Type	Modification enzyme	Site	Biological Functions	Metabolites	Metabolic pathways	Ref.
Phosphorylation	AMPK	T311	Disrupts EZH2 interaction with SUZ12, suppresses PRC2 HMTase activity and releases target genes (OLIG2, SOX17, GATA6), resulting in cell cycle arrest and cell differentiation	ATP	Glucose metabolism (glycolysis, etc.)	[78]
	AKT	S21	Suppresses EZH2 HMTase activity and releases target genes (HOXA9), promoting tumor progression	ATP	Energy metabolism	[79]
		S21	Inhbits EZH2 HMTase activity and releases target genes (IGF1, BCL2, HIF1A), leading to the MM cell drug resistance	ATP	Energy metabolism	[83]
		S21	Activates EZH2 as a coactivator for critical transcription factors including the androgen receptor, promoting tumor cells growth	ATP	Energy metabolism	[31]
		S21	Facilitates EZH2-mediated STAT3 methylation and enhances EZH2-STAT3 interaction and STAT3 activity, which accelerates GSC self-renewal and glioblastoma tumor process	ATP	Energy metabolism	[28]
Acetylation	PCAF	K348	Enhances EZH2 stability and function and the migration and invasion ability of lung cancer	Acetyl-CoA	Fatty acid β-oxidation, TCA cycle, pyruvate and acetate metabolism	[94]
O-GlcNAcylation	OGT	S75	Enhances EZH2 stability and function, contributing to tumorigenesis	UDP-GlcNAc	Glucose, amino acids, fatty acids and nucleotide metabolism	[98]
		S73, S76, S84, T313, S729	Enhance EZH2 stability and function, promoting cancer progression			[99]
		——	Promotes the migration and invasion of advanced colorectal cancer by enhancing EZH2 stability and activity			[100]

## Combined intervention of epigenetic regulation and metabolic processes and its potential in tumor treatment

Cancer cells adapt to the tumor microenvironment due to interaction between epigenetic regulation and metabolic activities. Currently, many drugs have been used to interfere with epigenetic regulation or metabolic activities. However, this approach is usually ineffective singularly or prone to drug resistance. A combination of interventions in epigenetic regulation and metabolic activities of tumor cells might help achieve better therapeutic effects.

### EZH2 inhibitors alone are not effective

As mentioned previously, many highly efficient and selective EZH2 inhibitors such as EPZ005687, EI1, GSK343, and GSK126 have been discovered [103]. EPZ-6438 and GSK126 have demonstrated some efficacy in hematological malignancies, including DLBCL carrying the EZH2 Y641 mutant and FL [18, 19]. However, cells of various lymphomas often acquire resistance after treatment with EZH2 inhibitors [104]. Additionally, the antitumor effect of EZH2 inhibitors alone is not optimally efficacious against EZH2 overexpressed solid tumors. For example, it could lead to drug resistance of EZH2 inhibitors in gliomas and melanomas bearing simultaneous mutations in Ras and SWI/SNF [105, 106].

Recently, a triple combination of EZH2, BRD4, and MAPK pathway inhibitors has improved the efficacy of solid tumor models with abnormally high levels of EZH2. Researchers have found that blocking H3K27me3 using EZH2 inhibitors had resulted in the upregulation of p300/CBP-mediated H3K27ac leading to the activation of oncogenes such as *WNT*. Therefore, a combined strategy with the H3K27ac synergistic inhibitor (JQ1) was adopted to improve the efficacy of the EZH2 inhibitor (EPZ-6438), through its singular application. However, some cancers (such as those involving the SMMC-7721 cell line) can still activate oncogenic signaling pathways (KRAS, AKT, etc.). Furthermore, the study has found that a combination of all three inhibitors in synergism with those of the MAPK pathway could inhibit the proliferation of resistant cells at the end [107]. Thus, the development of multi-mechanical combined medication for EZH2 may be an effective strategy to improve antitumor treatment.

### Potential value of combined intervention of EZH2 and tumor metabolic activities

EZH2 inhibitors can weaken drug resistance caused by tumor metabolic activities to a certain extent. For example, in solid tumors, the vascular system is underdeveloped, resulting in hypoxia and glutamine deficiency [108, 109]. This promotes the dedifferentiation of cancer cells and induces the development of drug resistance, leading to poor clinical outcomes [110]. Regulating regional glutamine levels in tumors is still challenging. However, EZH2 inhibitors (such as EPZ005687 and GSK126) may directly block H3K27 hypermethylation, activate pro-differentiating genes, and inhibit the expressions of dedifferentiation markers [108]. Therefore, targeted epigenetic regulation can weaken the carcinogenic properties caused by tumor metabolism.

Regulating tumor cell metabolism may downregulate EZH2 activity in concert with EZH2 inhibitors to intervene with tumor progression. Being a sensor of energy metabolism, AMPK is activated during conditions of energy stress (such as glucose deprivation and glycolysis block) and phosphorylates EZH2. EZH2 phosphorylation releases PRC2/EZH2-mediated gene silencing and facilitates the expression of target genes to inhibit the survival of tumor cells [78]. This finding suggests that AMPK agonists could be sensitizers for anticancer treatment of EZH2-targeted drugs. Targeting tumor metabolism and EZH2 in combination could be a new way in resolving the poor therapeutic efficacy of EZH2 inhibitors alone.

## Conclusions and perspective

Briefly, epigenetic regulation and metabolic alteration mediated by EZH2 demonstrate a synergistic effect in cancer cells. Additionally, the modifications of EZH2 and metabolic alterations in cancer cells are highly intertwined, respectively. For example, K348 acetylation of EZH2 decreases its phosphorylation at T345 and T487 and increases its stability. Additionally, frequent competition between O-GlcNAcylation and phosphorylation for occupancy at serine/threonine sites is observed. Therefore, it should be considered holistically to determine the interaction and respective characteristics with epigenetics and metabolism. It is also noteworthy that each tissue has specific characteristics in epigenetic landscapes and metabolic profiles. Thus, different combinations of EZH2 inhibitors and metabolic regulators need to be based on different scenarios.

Recently, the FDA has approved tazemetostat (Tazverik; Epizyme), the first EZH2 inhibitor to receive the agency’s nod for the treatment of adults and adolescents aged ≥ 16 years with locally advanced or metastatic epithelioid sarcoma not eligible for complete resection [1, 2]. Meanwhile, Epizyme is currently conducting a comprehensive development program for tazemetostat in three aspects, which are as follows: (1) tazemetostat is being investigated as a single-agent therapy in cancers directly dependent on EZH2 activity, including non-Hodgkin lymphoma, INI1-negative tumors, and synovial sarcoma, and (2) tazemetostat is also being evaluated as a combination with R-CHOP (a chemotherapy regimen) for newly diagnosed elderly, high-risk patients with DLBCL, and (3) tazemetostat is also combinated with Tecentriq (anti-PD-L1 cancer immunotherapy) for patients with relapsed or refractory DLBCL. Epigenetic drugs including tazemetostat are often used as a combination with other drugs in clinical trials, such as romidepsin (HDAC inhibitor) and etoposide (a chemotherapy regimen) for the treatment of patients with relapsed or refractory peripheral T cell lymphoma [111]. At present, the effects of different combinations of EZH2 inhibitors and other drugs are being investigated in antitumor therapy. For example, inhibition of EZH2 expression in T cells increases the effectiveness of anti-CTLA-4 therapy in MB49 (bladder) and B16-F10 (melanoma) tumor-bearing C57BL/6 mice [112]. And GSK126 was found to significantly promote chemotherapeutic drug (epirubicin and mitomycin C)-induced genotoxicity and increased HepG2 cell chemosensitivity [113]. Therefore, to improve the limited effectiveness of EZH2 inhibitors in clinical treatment, the optimization of different combination including the metabolic regulators, immunotherapy, radiotherapy, and chemotherapy are the promising detection in the near future.

## Data Availability

Not applicable.

## Abbreviations

ACLY: ATP-citrate lyase
ACSS2: Acyl-CoA synthetase short-chain family member 2
AMPK: AMP-activated protein kinase
APOE: Apolipoprotein E
AR: Androgen receptor
Bax: B cell lymphoma 2 (Bcl-2) associated X protein
BCAA: Branched-chain amino acids
BCAT: Branched-chain aminotransferases
BCKAs: Branched-chain α-keto acids
C/EBPα: CCAAT/enhancer binding protein-α
CDK: Cyclin-dependent kinase
ChIP-seq: Chromatin immunoprecipitation sequencing
CRC: Colorectal cancer
CRPC: Castration-resistant prostate cancer
DDR: DNA damage repair
DLBCL: Diffuse large B cell lymphoma
DNMT: DNA methyltransferase
ECAR: Extracellular acidification rate
ENO2: Enolase2
EZH2: Enhancer of zeste homolog 2
FASN: Fatty acid synthase
FL: Follicular lymphoma
GBM: Glioblastoma
GCB: Germinal central B cell
GLUT1: Glucose transporter 1
GSC: Glioblastoma stem-like cells
H2BS36: Phosphorylate H2B at serine 36
H3K27me3: Histone 3 lysine 27 trimethylation
HK1/2: Hexokinase 1/2
HMT: Histone methyltransferase
K348: lysine348
LAT1: L-type amino acid transporter 1
LC-ESI-MS: Liquid chromatography-electrospray ionization-mass spectrometry
LSC: Leukemia stem cells
MAPK: Mitogen-activated protein kinase
MAT: Methionine adenosyl transferase
MDS: Myelodysplastic syndrome
MDS/MPN: Myelodysplastic/myeloproliferative neoplasms
MPN: Myeloproliferative tumor
OGA: O-GlcNAcase
O-GlcNAcylation: O-linked N-acetylglucosamine modification
OGT: O-GlcNAc transferase
OXPHOS: Oxidative phosphorylation
PCa: Prostate cancer
PcGs: Polycomb group proteins
PCK2: Phosphoenolpyruvate carboxykinase
PDC: Pyruvate dehydrogenase complex
PDK1: Pyruvate dehydrogenase kinase 1
PFK1/2: Phosphofructokinase 1/2
PGC-1α: Peroxisome proliferator-activated receptor gamma coactivator 1-α
PPARγ: Proliferator-activated receptor-γ
PRC2: Polycomb repressive complex 2
pS21-EZH2: Phosphorylation of EZH2 at Ser21
pT311-EZH2: Phosphorylation of EZH2 at Thr311
PTMs: Post-translational modifications
RNAPII: RNA polymerase II
RORα: RAR-related orphan receptor α
RPS6KB1: Ribosomal protein S6 kinase B1
RXRα: Retinoid X receptor-α
S6K1: S6 kinase 1
S729: Serine 729
S73: Serine 73
S75: Serine 75
S84: Serine 84
SAH: S-adenosyl-l-homocysteine
SAM: S-adenosine methionine
Ser: Serine
SREBP-1c: Sterol regulatory element-binding protein-1c
T313: Threonine 313
TCA cycle: Tricarboxylic acid cycle
TERT: Telomerase reverse transcriptase
Thr: Threonine
α-KG: α-ketoglutarate
